# Pulmonary 99mTc-HMDP uptake correlates with restrictive ventilatory defects and abnormal lung reactance in transthyretin cardiac amyloidosis patients

**DOI:** 10.1186/s12931-022-01995-x

**Published:** 2022-03-27

**Authors:** Astrid Monfort, Alexia Rivas, Rishika Banydeen, Jocelyn Inamo, Karim Farid, Remi Neviere

**Affiliations:** 1 Department of Cardiology, CHU Martinique (University Hospital of Martinique), 97200 Fort-de-France, France; 2 Department of Nuclear Medicine, CHU Martinique (University Hospital of Martinique), 97200 Fort-de-France, France; 3 Department of Clinical Research, CHU Martinique (University Hospital of Martinique), 97200 Fort-de-France, France; 4 Cardiovascular Research Team EA7525, Université des Antilles (University of the French West Indies), 97200 Fort-de-France, France; 5 INSERM U1144, Université de Paris, 75006 Paris, France

**Keywords:** Transthyretin, Cardiac amyloidosis, ^99m^Tc-HMDP, Lung function tests, Aerobic capacity

## Abstract

**Background:**

Pulmonary involvement in individuals with transthyretin cardiac amyloidosis is unclear. The aim of this study was to quantify ^99m^Tc-hydroxy methylene diphosphonate (HMDP) lung retention in hereditary transthyretin (ATTRv) cardiac amyloidosis patients and to relate tracer uptake intensity to pulmonary function and aerobic capacity.

**Methods:**

We prospectively enrolled 20 patients with biopsy-proven ATTRv cardiac amyloidosis and 20 control subjects. Cardiac involvement was confirmed by echocardiography and nuclear imaging using ^99m^Tc-HMDP. Semi-quantitative analysis of the heart, rib and lung retention was assessed using a simple region of interest technique. Pulmonary function was evaluation by the means of whole-body plethysmography, diffusing capacity of the lung for carbon monoxide, forced oscillation technique and cardiopulmonary exercise testing.

**Results:**

Pulmonary tracer uptake estimated by lung to rib retention ratio was higher in ATTRv amyloidosis patients compared with control subjects: median 0.62 (0.55–0.69) vs 0.51 (0.46–0.60); p = 0.014. Analysis of relation between lung ^99m^Tc-HMDP retention and pulmonary function parameters shown statistically significant correlations with total lung volume (% predicted), lung reactance (X_rs_ 5 Hz) and peak VO_2_, suggesting total lung capacity restriction impaired elastic properties of the lung and poor aerobic capacity.

**Conclusion:**

Our study suggests that some grade of pulmonary retention of ^99m^Tc-HMDP may occur in patients with cardiac ATTRv amyloidosis, which can elicit deleterious effects on patient’s lung function and aerobic capacity.

**Supplementary Information:**

The online version contains supplementary material available at 10.1186/s12931-022-01995-x.

## Introduction

Systemic amyloidosis is a rare disease resulting from extracellular deposition of amyloid fibrils such as monoclonal immunoglobulin light chains (AL) or transthyretin (ATTR), either in its acquired (ATTRwt) or hereditary (ATTRv) form [1–4]. In ATTRwt amyloidosis, transthyretin is a normal protein and the disease affects the elderly and primarily presents as heart failure [5]. In ATTRv amyloidosis, more than 120 mutations have been reported in the transthyretin gene with considerable phenotypic and geographical heterogeneity [4, 6]. The most common variant in ATTRv amyloidosis is Val122Ile (p.Val142Ile). In this genotype, accumulation of the mutated transthyretin has a clear multisystem tropism involving the heart and the peripheral nervous system [3, 7, 8].

Pulmonary manifestations of systemic amyloidosis are relatively common, whilst rarely symptomatic [7, 9, 10]. In AL amyloidosis, lung deposit of the amyloid protein can appear in different presentations including nodular pulmonary amyloidosis, diffuse alveolar-septal amyloidosis and tracheobronchial amyloidosis [7, 10]. In ATTR amyloidosis, lung involvement is considered to be rare, but histopathological findings such as amyloid deposit in the alveolar walls, pleural effusions, and lung nodules, have been reported in post mortem studies and case reports [11–16]. Likewise, scintigraphy studies using ^99m^Tc-phosphate derivatives to diagnose cardiac amyloidosis have consistently revealed some grade of lung retention in ATTR and AL amyloidosis [17–21]. New amyloid radiotracers which are well-established for imaging beta amyloid are emerging as promising tools to evaluate other forms of systemic amyloid deposition. In line, lung uptake using ^18^F-florbetapir and ^18^F-florbetaben in systemic amyloidosis, have described mainly in cardiac amyloidosis [22–27].

The clinical significance of amyloid deposits in the lung of patients with ATTR cardiac amyloidosis remains unclear and warrants close follow-up of pulmonary function. The aim of this study was to quantify ^99m^Tc-hydroxy methylene diphosphonate (HMDP) lung retention in ATTRv cardiac amyloidosis patients and to relate tracer uptake intensity to aerobic capacity, pulmonary function assessed by body plethysmography, diffusion capacity for carbon monoxide, and elastic properties of lung periphery assessed by impulse oscillometry.

## Materials and methods

### Ethics and patients

The study was conducted in accordance with the amended Declaration of Helsinki (http://www.wma.net/en/30publications/10policies/b3/). Informed consent was obtained from all patients and an Institutional Review Board (IRB) approved all study procedures (IRB 00006477, APHP, France). Written informed consent was obtained from all patients. All the procedures and their risks were explained to the patients, who confirmed their verbal informed consent to enter the study at the time when referred to the cardiovascular department for routine functional evaluation.

The study was carried out at the Department of Cardiology, Martinique University Hospital, France from September 2018 to May 2021 in consecutive patients with known familial transthyretin amyloidosis mutation carriers (ATTRv). The diagnostic of systemic transthyretin amyloidosis was confirmed in all patients by histological demonstration of amyloid fibrils in salivary duct gland, subcutaneous adipose tissue or endomyocardial biopsies. All patients had echocardiography evaluation according to American Society of Echocardiography recommendations. Serum high-sensitivity troponin and NT-proBNP were used as cardiac biomarkers.

### ^99m^TC-HMDP scintigraphy

Cardiac involvement was confirmed by nuclear imaging (General Electric Medical Systems SPECT gamma camera Discovery NM630 or D670) using ^99m^Tc-HMDP at intravenous dose of 8 MBq/kg. Images were processed four hours post-injection by a whole-body scan (anterior and posterior projections) and by a thorax SPECT. Scanner was not required. A senior nuclear physician interpreted the images, blinded to clinical data. GE’s Volumetrix MI software (GE Healthcare) was used for semi quantitative analysis. The investigator classified the patients according to the Perugini score classification (0, no cardiac uptake and normal bone uptake; 1, slight cardiac uptake less marked than bone uptake; 2, moderate cardiac uptake with attenuated bone uptake; and 3, strong cardiac uptake with slight/absent bone uptake). Semi-quantitative analysis of the heart, rib and lung retention was assessed using a simple region of interest (ROI) technique of the SPECT acquisitions. ROI measurements were performed in the lateral arch of the 3rd or 4th right rib (excluding chondro-costal cartilage) and in the right lung apex both on the same section plane, and in the heart at the lateral wall of the LV or septum. ROI measurements were also reported for control group. Heart-to-rib retention and lung-to-rib ratios were computed for each group. Because there are no normal values for ^99m^Tc-HMDP pulmonary retention, ATTRv patients were compared with age and sex matched control subjects declaring African ancestries who had no cardiac amyloidosis. These subjects were referred to the Department of Nuclear Medicine for ^99m^Tc-HMDP scan for prostate adenocarcinoma extension. Patients with costal metastases or fractures, and lung parenchymal abnormalities on chest computed tomography were excluded. These subjects were NYHA I and had no history of systemic hypertension, type 2 diabetes or pulmonary diseases such as chronic obstructive pulmonary disease.

### Pulmonary function and impulse oscillometry system

Pulmonary function tests were performed using the plethysmography and diffusing capacity of the lung for carbon monoxide (DL_CO_) (Master Screen Body Jaeger, Hoechberg, Germany) equipped with the software Lab Manager V5.32.0 (CareFusion, Hoechberg, Germany). For the quality of forced vital capacity (FVC), total lung volume (TLC) and forced expiratory volume in 1 s (FEV_1_) measurements, the American Thoracic Society/European Respiratory Society test criteria for acceptability and repeatability were respected; from each spirometry and lung volume at least three measurements were taken to assure reproducibility [28]. The pulmonary resistance and reactance (5–25 Hz) were measured using IOS (MasterScreen IOS, Viasys GmbH, Hoechberg, Germany). Reactance can be considered as the out of phase component of respiratory impedance, reflecting the balance between inertial and elastic properties of distensible airways. Typically, this is measured at 5 Hz (Xrs5) reflecting the combined effect of tissue elastance and inertance [29, 30].

### Cardiopulmonary exercise testing

Cardiopulmonary exercise testing was performed according to standardized procedures using an electromagnetic braked cycle ergometer, as recommended by the ATS/ACCP [31]. Breath-by-breath cardiopulmonary data (PowerCube-Ergo, Ganshorn Medizin Electronic GmbH, Niederlauer, Germany) were measured at rest, warm up and incremental exercise testing (10 W/minute) until exhaustion at a pedaling frequency of 60–65 revolutions/minute (rpm). Minute ventilation (V_E_), oxygen uptake (VO_2_), carbon dioxide output (VCO_2_) were recorded as concurrent 10-s moving averages, as was determined ventilation anaerobic threshold by the V-slope method. Patient effort was considered to be maximal if two of the following occurred: predicted maximal work is achieved, age-predicted maximal heart rate (HRmax) is achieved, ventilatory O_2_ equivalent VE/VO_2_ > 45 and respiratory exchange ratio (RER, i.e. VCO_2_/VO_2_ ratio) > 1.10, as recommended by the ATS/ACCP.

### Statistics

For all descriptive and inferential analyzes, assumption of normal data distribution was analyzed. Mean and standard deviations were reported for normally distributed variables and median and interquartile range for non-normally distributed variables. Categorical variables were presented as absolute values and percentages. The following tests were used for group comparisons: Pearson’s correlations were used to express correlations, and p values were calculated using a two-tailed t test. A p value of < 0.05 was considered statistically significant. Statistical analyses were done using SPSS®, version 26 (IBM).

## Results

Main characteristics of patients with ATTRv cardiac amyloidosis are summarized Table 1. All patients (n = 20) declared African ancestries and had transthyretin gene sequencing displaying either ATTR Val122Ile (p.Val142Ile) or Ile107Val (p.Ile127Val) mutations. All patients were symptomatic with NYHA functional class II or higher status.

**Table 1:** Baseline characteristics of transthyretin cardiac amyloidosis and control patients

	ATTRv patients (n = 20)	Controls (n = 20)	p value
*Clinical characteristics*			
Age, years	75 ± 7	74 ± 2	0.523
Male gender	18 (90)	20 (100)	0.487
BMI, kg/m^2^	25 ± 3	26 ± 7	0.561
Diabetes	4 (20)	2 (10)	0.661
Hypertension	8 (40)	7 (35)	1.000
Carpal tunnel	15 (75)	1 (5)	< 0.001
*Cardiovascular evaluation*			
NYHA III/IV	16 (80)	4 (20)	< 0.001
Systolic BP, mmHg	135 ± 21	133 ± 17	0.688
Heart rate, bpm	80 ± 13	75 ± 7	0.138
QS wave	10 (50)	0 (0)	< 0.001
Low QRS voltage	9 (45)	0 (0)	< 0.001
IVS thickness, mm	16 ± 3	10 ± 2^(n=12)^	< 0.001
EDLVD, mm	91 ± 36	78 ± 17^(n=12)^	0.251
LV mass, g/m^2^	170 ± 50	125 ± 20^(n=12)^	0.006
LVEF, %	50 ± 17	63 ± 12^(n=12)^	0.027
Cardiac index, L/min/m^2^	2.0 ± 0.4	3.5 ± 0.8^(n=12)^	< 0.001
LA diameter, mm	54 ± 12	38 ± 16^(n=12)^	0.003
E/Ea	17 ± 7	12 ± 6^(n=12)^	0.048
TDE, ms	133 ± 37	126 ± 16^(n=12)^	0.541
Systolic PAP, mmHg	39 ± 10	22 ± 12^(n=12)^	< 0.001
TAPSE, mm	21 ± 3	20 ± 2^(n=12)^	0.315
*Spirometry*			
FEV_1_, % predicted	66 ± 21	88 ± 12	< 0.001
FVC, % predicted	69 ± 16	91 ± 9	< 0.001
FEV_1_/FVC, %	75 ± 9	72 ± 12	0.377
*Biological parameters*			
eGFR, mL/min/1.72 m^2^	75 (56–106)^(n=17)^	55 (45–98)^(n=19)^	< 0.001
Cardiac troponin T, ng/L	97 (48–295)^(n=15)^	na	
NT-proBNP, ng/L	2998 (1408–3720)^(n=12)^	na	
Val122Ile (p.Val142Ile) mutation	15 (75)	na	
Ile107Val (p.Ile127Val) mutation	5 (25)	na	
*Scintigraphy*			
Heart-to-contralateral lung ratio	1.89 ± 0.46	0.98 ± 0.33	< 0.001
Apex lung ROI/(rib ROI-lung ROI)	1.30 ± 0.65	0.88 ± 0.24	0.010

Results are presented as mean ± standard deviation or median and interquartile ranges (IQR 25–75%) for quantitative variables, and as absolute value (percentage) for categorical variables. (na) indicates non applicable. (^n^) indicates sample size when different from 20

*ATTRv* hereditary or mutated transthyretin amyloidosis (v for variant); *BMI* body mass index; *NYHA*New York Heart Association (NYHA) classification; *BP* blood pressure; bpm beats per minute; *IVS* interventricular septum thickness; *EDLVD* End diastolic left ventricular diameter; *LV* left ventricle; *LVEF* left ventricular ejection fraction; *LA* left atrium; *E/Ea* peak of pulsed Doppler E wave/average peak of annulus tissue Doppler imaging E′ waves; *TDE* E deceleration time; *PAP* pulmonary arterial pressure; *ROI* region of interest; *TAPSE* tricuspid annular systolic excursion; *eGFR* estimated glomerular filtration rate; *NT-proBNP* N-terminal pro B-type Natriuretic Peptide

Analysis of ^99m^Tc-HMDP scintigraphy for cardiac uptake revealed that five patients (25%) had a Perugini grade 3, twelve (60%) grade 2, 2 (10%) had grade 1 and only one (5%) had no cardiac uptake. Pulmonary retention of ^99m^Tc-HMDP was higher in ATTRv amyloidosis patients compared with age and sex matched control subjects: lung to rib retention ratio (median interquartile (25–75%) range) of 0.62 (0.55–0.69) vs 0.51 (0.46–0.60); p = 0.014, respectively. Lung to rib retention ratio was not significantly different between each Perugini grade of ^99m^Tc-HMDP scan (p = 0.886).

Spirometry, plethysmography and impulse oscillometry (IOS) measurements are summarized Table 2. Considering lung function as normal: FEV_1_/FVC ≥ 0.70 and FEV_1_ ≥ 80% predicted) or restricted (FEV_1_ or < 80% predicted normal) in the absence of airflow obstruction (FEV_1_/FVC ≥ 0.70), fourteen patients (70%) displayed ventilatory restrictive pattern. In the subgroup of TTRv amyloidosis patients (n = 11) able to perform single breath-hold maneuver (10 s. apnea) allowing measures of diffusing capacity of the lung for carbon monoxide, 55% of cases displayed abnormal diffusing capacity of the lung (DL_CO_), while K_CO_ was normal. Predicted values of cardiopulmonary exercise testing (CPET) in patients able to performed submaximal exercise (n = 13) are presented Table 2. Aerobic capacity was significantly reduced in ATTRv amyloidosis patients, whereas parameters suggesting abnormal cardiac (i.e., low O_2_ pulse, increased V_E_-VCO_2_ slope) or pulmonary (i.e., ventilatory reserve), response were impaired as well (Table 2).

**Table 2:** Lung function and cardiopulmonary exercise variables of transthyretin cardiac amyloidosis patients (n = 20)

		n (%) of patients with value < 75% predicted
FEV_1_, liter	1.9 ± 0.6	
FEV_1_, % predicted	66.0 ± 21.0	14 (70)
FVC, liter	2.4 ± 0.7	
FVC, % predicted	69.0 ± 16.0	14 (70)
FEV_1_/FVC, %	75.0 ± 9.0	1 (5)
TLC, liter	5.0 ± 1.1	
TLC, % predicted	68.0 ± 17.0	14 (70)
R_rs_(5), kPa L^−1 ^s^−1^	0.4 ± 0.1	
X_rs_(5), kPa L^−1^ s^−1^	− 0.1 ± 0.1	
DL_CO_ (%)^n=11^	71.0 ± 17.0	6 (55)
K_CO_ (%)^n=11^	100.0 ± 16.0	0 (0)
Peak VO_2_, mL kg^−1^ min^−1 n=13^	15.0 ± 2.4	
Predicted VO_2_, % ^n=13^	65.0 ± 19.0	11 (85)
Peak RER ^n=13^	1.2 ± 0.1	
Ventilatory reserve, % ^n=13^	32.0 ± 19.0	5 (38)
Peak O_2_ pulse, % ^n=13^	75.0 ± 16.0	6 (46)
Peak systolic pressure, mmHg ^n=13^	175.0 ± 32.0	
Peak heart rate, % maximal predicted^n=13^	83.0 ± 14.0	

Results are presented as mean ± standard deviation and as absolute number and percent (n (%)). (^n^) indicates sample size when different from 20

*FEV*_*1*_ forced expiratory volume in 1 s; *FVC* forced vital capacity; *TLC* total lung capacity; *R*_*rs*_*(5)* respiratory system resistance at 5 Hz; *X*_*rs*_*(5)* respiratory system reactance at 5 Hz; *DL*_*CO*_ diffusion capacity of the lung for carbon monoxide; *K*_*CO*_ transfer coefficient of the lung for carbon monoxide; *VO*_*2*_ oxygen uptake; *RER* respiratory exchange ratio

**Fig. 1:**
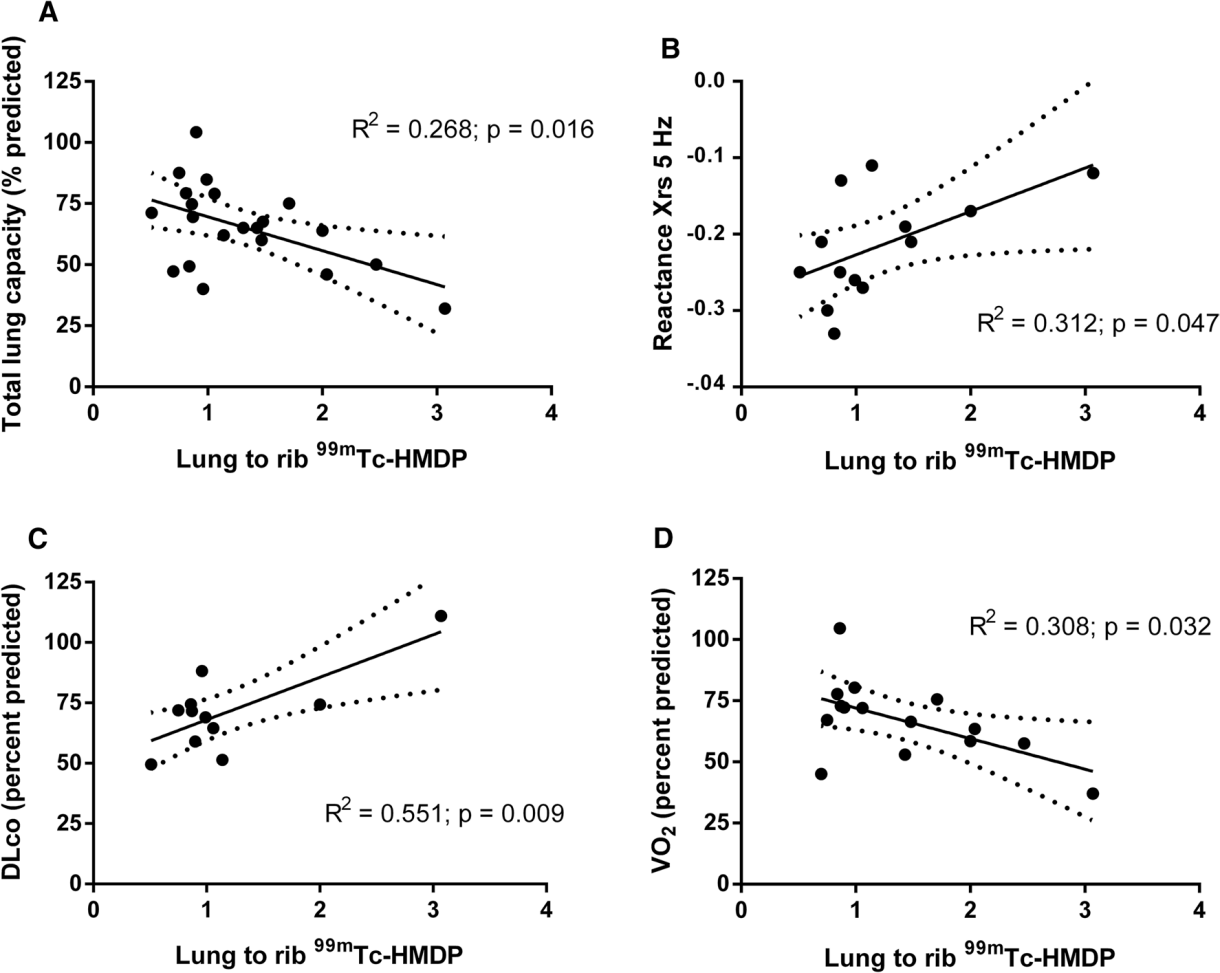
Simplified quantitative metrics of ^99m^Tc-HMDP in the lungs and their correlation with **A** total lung capacity; **B** lung reactance; **C** transfert coefficient for carbon monoxide (K_CO_); **D** peak oxygen uptake (VO_2_)

Next, relation between lung ^99m^Tc-HMDP retention and pulmonary function was investigated. When comparing lung to rib ^99m^Tc-HMDP retention ratio to parameters of pulmonary function, a statistically significant correlation was found with TLC (% predicted), suggesting that ^99m^Tc-HMDP lung retention increase was associated with reduction in lung volume (Fig. 1A). Lung retention of ^99m^Tc-HMDP was correlated with X_rs_ 5 Hz (Fig. 1B). As more elastic properties of the lung are more negative X_rs_ 5 Hz, the latter correlation suggested that increased ^99m^Tc-HMDP lung retention was associated with impaired elastic properties of the lung. In the subgroup of ATTRv amyloidosis patients (n = 11) able to perform single breath-hold maneuver (10 s. apnea) for diffusing capacity of the lung for carbon monoxide, we found that lung retention of 99mTc-HMDP was correlated with K_CO_ (Fig. 1C). In patients (n = 13) able to perform submaximal exercise, reduced aerobic capacity was associated with high pulmonary uptake of 99mTc-HMDP (Fig. 1D). No statistically significant correlations were found toward other cardiopulmonary functional parameters.

## Discussion

New findings of this study are twofold. Firstly, we observed that lung retention of ^99m^Tc-HMDP was higher in ATTRv cardiac amyloidosis patients compared with age and sex matched control subjects. Secondly, increase ^99m^Tc-HMDP lung uptake was associated with abnormal cardiopulmonary manifestations including reduction in lung volume, impaired elastic properties of the lung and poor aerobic capacity.

Imaging of amyloid deposits is challenging. Nuclear medicine has the advantage of providing noninvasive whole-body scanning for the detection of systemic forms. Our results are consistent with previous studies using ^18^F-florbetapir positron emission tomography/computed tomography (PET/CT) and ^18^F-florbetaben positron emission tomography/magnetic resonance imaging (PET/MRI) demonstrating extra cardiac uptake in patients with cardiac amyloidosis [22–27]. In particular, involvement of the lungs has been well imaged by ^18^F-florbepatir, suggesting pulmonary amyloid deposits [24, 25]. Increased lung uptake of ^18^F-florbetapir was further related to specific binding to pulmonary amyloid deposits as the absence of ^11^C-acetate lung uptake argued against the confounding effects of pulmonary accumulation of tracers related to elevated left atrial pressures and slower transit time through the pulmonary vascular bed related to myocardial dysfunction associated with cardiac amyloidosis. Overall, it was concluded that the opposite finding of minimal ^11^C-acetate lung uptake in patients with elevated ^18^F-florbetapir lung uptake likely represents lung amyloidosis [25].

Whereas lung amyloidosis is a well-established manifestation of AL systemic amyloidosis [7, 10], only rare pathological post-mortem findings, case reports and nuclear imaging studies suggest involvement of the lung in both ATTRwt and ATTRv systemic amyloidosis [11–16]. Likewise, limited information is available regarding functional consequences of lung amyloid deposits [10]. Consistent with amyloid fibrillogenesis processes in several organs, amyloid fibrils accumulate within the lung interstitium in the form of branching filaments, which may favor lung volume restriction and parenchymal stiffness, as well as gas diffusing capacity impairment. Interestingly, our study suggested that more lung ^99mTc^-HMDP uptake was more impaired pulmonary function. We found that a large proportion of patients with ATTRv cardiac amyloidosis had restrictive ventilatory pattern and impaired diffusing capacity of the lung for carbon monoxide. Our study also suggests that lung retention of ^99m^Tc-HMDP might be responsible, at least in part, for total lung capacity restriction and impaired elastic properties of the lung. That high ^99m^Tc-HMDP lung uptake levels correlated with above normal lung diffusing capacity value is confusing because accumulation of amyloid fibrils is expected to alter gas diffusion through the alveolar-capillary membrane. Possible explanation for this unexpected result is the increase rate of carbon monoxide removal from the alveolar secondary to pulmonary capillary blood accumulation [32], which may be related to myocardial dysfunction in ATTRv cardiac amyloidosis. Complete evaluation of functional capacity in ATTRv amyloidosis patients was performed using cardiopulmonary exercise testing (CPET). We found that the reduction of aerobic capacity was associated with intense pulmonary uptake of ^99m^Tc-HMDP. No associations were found between lung ^99m^Tc-HMDP uptake and CPET-derived parameters known to distinguish between cardiac or respiratory causes of functional impairment.

Our study has several limitations. Patients were prospectively enrolled using strict inclusion and exclusion criteria of systemic transthyretin amyloidosis. A limitation of the study is the lack of histological confirmation as gold standard to identify pulmonary amyloidosis. Of note, lung and pleural biopsies are not routinely performed in ATTR patients and a correlative study with pulmonary histology is lacking. It would seem relevant to achieve a scan in addition to the tomo-scintigraphy to study lung injuries, especially when the scintigraphy is requested in cardiac decompensation stage. The scanner should also be used to visualize lesions that could increase the density of the lung parenchyma and therefore its uptake in scintigraphy, such as atelectasis or collapse. That ^99m^Tc-HMDP lung uptake actually represents lung amyloid fibrils deposit in our patients warrant further investigation. Other limitation was that this study is limited to patients with known cardiac amyloidosis and only 20 ATTR patients were included. This low sample size is mainly explained by the fact that cardiac amyloidosis is a rare disease even in expert centers evaluating cardiopulmonary function in cardiac amyloidosis patients.

In conclusion, our study suggests that some grade of pulmonary retention of ^99m^Tc-HMDP may occur in patients with cardiac ATTR amyloidosis, which can elicit deleterious effects on patient’s lung function and aerobic capacity.

### Supplementary Information

**Additional file 1.** Raw data of patients with cardiac transthyretin amyloidosis.

## Data Availability

Raw data are provided in the Additional file 1.

## Declarations

### Declaration of transparency

The lead author NEVIERE Remi affirms that this manuscript is an honest, accurate, and transparent account of the study being reported; that no important aspects of the study have been omitted; and that any discrepancies from the study as planned (and, if relevant, registered) have been explained. Signed by: NEVIERE Remi *The manuscript’s guarantor.
